# Immunomodulators, Biologics, and 5-ASA for Inflammatory Bowel Disease and Major Adverse Cardiovascular Events in Older Adults

**DOI:** 10.1001/jamanetworkopen.2026.9091

**Published:** 2026-04-29

**Authors:** Qi Jian, Naueen A. Chaudhry, Fanxing Du, Ellen Zimmermann, Tianze Jiao

**Affiliations:** 1 Department of Pharmaceutical Outcomes and Policy, College of Pharmacy, University of Florida, Gainesville; 2 Division of Gastroenterology, Hepatology and Nutrition, Department of Medicine, College of Medicine, University of Florida, Gainesville; 3 Center for Drug Evaluation and Safety, College of Pharmacy, University of Florida, Gainesville

## Abstract

**Importance:**

Patients with inflammatory bowel disease (IBD) are at increased risk of major adverse cardiovascular events (MACE), driven by chronic inflammation, endothelial dysfunction, and use of certain therapeutic regimens. Whether different IBD treatments mitigate this risk remains unclear.

**Objective:**

To examine the association of the use of immunomodulators or biologics vs 5-aminosalicylic acid (5-ASA) with risk of MACE among older patients with IBD.

**Design, Setting, and Participants:**

This comparative effectiveness study was conducted among a 15% Medicare claims sample of patients with IBD aged 65 years or older was identified, with entry defined by the first prescription of immunomodulators, biologics, or 5-ASA between January 1, 2012, and December 31, 2020. Follow-up extended to the outcome, switch or discontinuation of study drug, the study’s administrative end, or up to 3 years, whichever occurred first. Propensity score matching at a 1:3 ratio balanced demographics, comorbidities, and medication use between groups. Data analyses were conducted between January and September 2025.

**Exposures:**

Exposures were use of immunomodulators or biologics compared with 5-ASA, identified using National Drug Codes and Healthcare Common Procedure Coding System procedure codes.

**Main Outcomes and Measures:**

The primary outcome was time to the first emergency department or inpatient visit caused by a MACE, defined as myocardial infarction, stroke, or all-cause mortality. Hazard ratios (HRs) and 95% CIs for MACE risk with immunomodulators or biologics vs 5-ASA were estimated using Cox proportional hazards models.

**Results:**

A total of 16 387 patients (mean [SD] age, 74.73 [6.79] years; 9861 [60.18%] female) were included in the analysis. In the immunomodulators vs 5-ASA and biologics vs 5-ASA cohort, the mean (SD) ages were 74.05 (6.43) years and 73.68 (6.01) years, respectively, after matching. Both cohorts had more female participants (2580 [58.85%] and 1780 [58.09%], respectively). Baseline comorbidities were mostly balanced between groups. Compared with 5-ASA, there was no statistically significant difference in the risk of MACE for immunomodulators (HR, 0.84 [95% CI, 0.61-1.17]) or biologics (HR, 0.86 [95% CI, 0.59-1.24]), although point estimates were below 1.

**Conclusions and Relevance:**

In this comparative effectiveness study of Medicare beneficiaries with IBD, there was no statistically significant difference in MACE risk between those who used immunomodulators or biologics vs 5-ASA.

## Key Points

**Question:**

Is type of inflammatory bowel disease (IBD) treatment associated with risk of major adverse cardiovascular events (MACE) in Medicare patients with IBD?

**Findings:**

In this comparative effectiveness study of 16 387 Medicare beneficiaries with IBD, there were no statistically significant differences in MACE events over up to 3 years of follow-up between patients treated with immunomodulators or biologics vs those treated with 5-aminosalicylic acid (5-ASA).

**Meaning:**

This comparative effectiveness study found that use of immunomodulators or biologics was not associated with increased risk of MACE in older adults with IBD compared with 5-ASA therapy.

## Teaser

This comparative effectiveness study examines the association of inflammatory bowel disease treatment with immunomodulators or biologics vs 5-aminosalicylic acid with risk of major adverse cardiac events in older adults.

## Introduction

Ulcerative colitis (UC) and Crohn disease (CD), collectively known as inflammatory bowel disease (IBD), affect approximately 3 million people in the US alone.^1^ Current IBD treatment guidelines aim to control inflammation and maintain remission through agents such as corticosteroids, 5-aminosalicylates (5-ASA), immunomodulators, biologics, and small-molecule drugs.^2,3,4^ For mild to moderate colonic inflammation, 5-ASAs are often used as the first-line therapy and act locally on the bowel mucosa to suppress inflammation.^5^ For other patients, including those with moderate to severe IBD or those do not respond to 5-ASA therapy,^4^ biologics, including anti–tumor necrosis factor (TNF) therapy, anti–interleukin (IL) 12 and 23 therapy, and anti-integrin therapy, are the standard of care, targeting specific immune pathways driving inflammation.^6,7,8^ Immunomodulators, such as tacrolimus and methotrexate, are mainly used alongside biologics to reduce immunogenicity for anti-TNF agents and as supplementary therapy in select patients,^9^ working by inhibiting T-cell proliferation and proinflammatory cytokines while promoting anti-inflammatory responses, like IL-10 and transforming growth factor β.^10^

Emerging data suggest that IBD is associated with a higher risk of cardiovascular disease (CVD).^11,12,13^ Patients with IBD have a 19% to 103% higher risk of heart failure,^14,15^ 52% to 126% increased risk of atrial fibrillation,^16,17^ 15% to 27% increased risk of myocardial infarction,^18,19^ and 27% higher risk of major adverse cardiovascular events (MACE).^20^ Chronic systemic inflammation, driven by elevated proinflammatory cytokines, like TNF-α, IL-1β, IL-6, and C-reactive protein, along with gut microbial dysbiosis and increased formation of neutrophil extracellular traps, promotes reactive oxygen species generation and endothelial dysfunction, ultimately contributing to atherosclerotic CVD^12,21^ and MACE.^22,23^ Given the inflammatory link, it is biologically plausible that effective control of systemic inflammation with immunomodulators or biologics could mitigate CVD due to their favorable effects on inflammatory pathways. However, existing data are mixed, with a network meta-analysis of randomized clinical trials reporting that biologics were associated with higher odds of MACE vs placebo.^24^ As a result, clinicians remain uncertain whether immunosuppressive regimens mitigate or exacerbate MACE.

This uncertainty is particularly significant for older adults with IBD, as they carry high baseline risk of MACE due to the combined effects of age, comorbidities, and chronic inflammation burden,^25^ while simultaneously facing increased risk of serious infection and malignant neoplasms associated with immunomodulator and biologic therapy.^26^ The lack of evidence and safety concerns specific to older adults may contribute to underutilization of immunomodulators and biologics in this population.^27^ Therefore, this study aims to bridge the knowledge gap by examining the association between the use of immunomodulators or biologics therapies and the risk of MACE among older adults with IBD.

## Methods

This comparative effectiveness study was approved by the University of Florida institutional review board, and an appropriate data use agreement was in place prior to data abstraction. This study was exempt from review and the requirement of informed consent by the University of Florida institutional review board under Category 4 (secondary research without consent) of 45 CFR §160 and §164. This study was conducted and reported in accordance with the Strengthening the Reporting of Observational Studies in Epidemiology (STROBE) reporting guideline.

### Data Source, Study Design, and Eligibility

We used a 15% random sample from Medicare fee-for-service beneficiaries from Medicare Parts A (inpatient services), B (outpatient services), and D (prescription medications) claims, spanning from January 1, 2012, to December 31, 2020. Medicare is a federal health insurance program in the US that provides coverage primarily to individuals aged 65 years and older, as well as to younger individuals with certain disabilities.

Patients entered the base cohort at the date of their first inpatient or second outpatient visit with an IBD diagnosis, including CD and UC, identified by *International Classification of Diseases, Ninth Revision, Clinical Modification* (*ICD-9-CM*) or *International Statistical Classification of Diseases, Tenth Revision, Clinical Modification* (*ICD-10-CM*) codes. Thereafter, they entered the study cohort when they received the first prescription of 5-ASA, immunomodulators, or biologics, with the prescription fill date defined as the index date. We excluded patients who were younger than 65 years; experienced the outcome of interest within 12 months preceding the index date; switched IBD medications on treatment class within 12 months preceding the index date; did not have continuous enrollment in Medicare Parts A, B, and D between base cohort entry date and index date; or used related biologics prior to their approval for IBD.

### Exposure Ascertainment

Two separate exposure cohorts were defined: patients initiating immunomodulators vs 5-ASA and patients initiating biologics vs 5-ASA, with 5-ASA serving as the reference group in both comparisons. We used generic name from Medicare Part D and related Healthcare Common Procedure Coding System codes (mainly for biologics) to extract the prescription patient received. Each patient’s exposure was classified based on the first prescription they received within these drug categories. Healthcare Common Procedure Coding System codes did not provide days of supply, so the days of supply for each medication were defined according to its label-based dosing interval. We then calculated the proportion of days covered using related prescriptions in Part D to assess refill patterns and determine the corresponding grace period for each medication, which was set equal to the observed refill interval.

### Study Outcome

The primary outcome was the time to the first emergency department or inpatient visit caused by MACE. MACE was defined as a composite of nonfatal myocardial infarction, nonfatal stroke, and all-cause mortality,^28^ extracted by *ICD-9-CM* and *ICD-10-CM* diagnosis codes.

### Follow-Up

A per-protocol analysis approach was used in the study. All eligible patients were followed up from the index date until the occurrence of the outcome of interest; switch or discontinuation of drug; death; disenrollment from Medicare Part A, B or D; or the end of the study period (defined as December 31, 2020, or the end of 3 years follow-up from the index date), whichever came first.

### Covariates

Patients baseline characteristics were measured during 1 year before the index date, including demographic characteristics (ie, age, sex, and race and ethnicity), lifestyle factors (ie, smoking status and alcohol-related disorder), comorbidities (eg, hypertension, type 2 diabetes, chronic kidney disease, hyperlipidemia, rheumatoid arthritis, organ transplant, obesity, gastroesophageal reflux disease, anemia, thyroid disorder, depression, anxiety, bipolar disorder, coronary artery disease, metabolic dysfunction–associated steatotic liver disease, IBD-related complications, cancer, and history of IBD-related surgical procedures), medication use (ie, steroids and statin), IBD subtype (UC or CD), cardiovascular risk score QRISK3, claims-based frailty score, Charlson Comorbidity Index, the year of the study cohort entry, and the duration of untreated IBD. Race and ethnicity were obtained from Medicare administrative claims data based on beneficiary enrollment records maintained by the Centers for Medicare & Medicaid Services, categorized as Asian, Black, Hispanic, North American Native, White, other, or unknown. In Medicare data, the “other” category does not represent a single self-identified racial or ethnic group, but rather a heterogeneous residual category. Although additional race/ethnicity categories (eg, American Indian, Asian, Hispanic) were introduced over time, incomplete updating of historical records and limitations in administrative data collection have resulted in some beneficiaries remaining classified as other. For analysis, participants were grouped as Black, White, or other, with the other category including beneficiaries recorded as Asian, Hispanic, North American Native, other, or unknown; categories were combined because of small sample sizes. Classifications were not assigned by the investigators.

### Statistical Analysis

Descriptive statistics were presented for baseline characteristics. Propensity scores (PSs) for treatment initiation were estimated using multivariable logistic regression, including all covariates. We applied 1:3 PS matching without replacement using a caliper of 0.25 times the SD of the logit of the score to balance covariates between the exposed (immunomodulator or biologic) and reference (5-ASA) groups. Covariate balance was assessed using standardized mean differences (SMD), with values less than 0.1 indicating negligible imbalance. Crude incidence rates and incidence rate ratios (IRRs) were calculated for each exposure group. Cox proportional hazards models estimated hazard ratios (HRs) and 95% CIs for time to MACE, with proportional hazards verified using Schoenfeld residuals. Variables with SMD of 0.1 or greater were additionally adjusted for in the Cox models.

To evaluate the robustness of our results, we conducted multiple sensitivity analyses. First, an intention-to-treat analysis assessed treatment outcomes regardless of treatment discontinuation or switching. Second, we refined the outcome definition by limiting MACE to myocardial infarction, stroke, and cardiovascular-related death, excluding death caused by other reasons. Deaths due to noncardiovascular causes were treated as competing events and handled using a cause-specific weighted hazard approach.^29^ Next, we applied latent periods of 1 and 6 months after treatment initiation to account for the induction time needed for 5-ASA and biologics to impact cardiovascular outcomes.^30,31,32^ Fourth, we conducted subgroup analyses for patients diagnosed with UC and CD separately, given that 5-ASA is more likely to be prescribed for patients with UC, who have a higher underlying CVD risk.^5,13^ Then, we applied inverse probability censoring weight to adjust for informative censoring, with weights truncated at the 5th and 95th percentiles to reduce the influence of extreme values. Lastly, to identify possible unmeasured confounding, we analyzed injury-related emergency department visits as a negative control outcome.

*P* values were 2-sided, and statistical significance was set at *P* < .05. Analyses were performed between January 2025 and September 2025 using SAS software version 9.4 (SAS Institute) and R software version 4.5.3 (R Project for Statistical Computing) for visualization.

## Results

**Figure 1.**
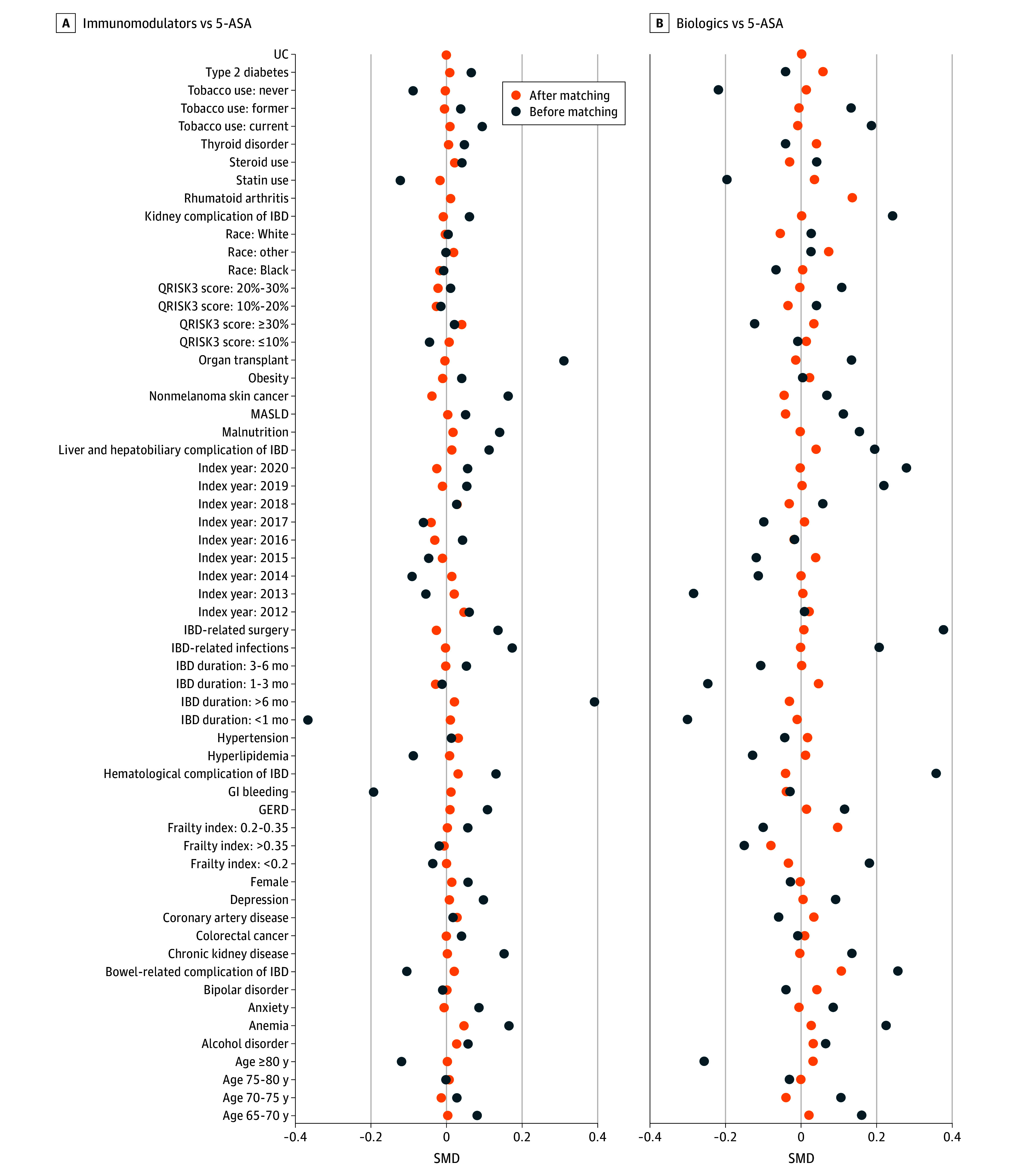
Dot Plots of Standardized Mean Differences (SMDs) for Baseline Characteristics Before and After Propensity Score Matching In the biologics vs 5-aminosalicylic acid (5-ASA) comparison, rheumatoid arthritis and bowel-related complications remained imbalanced and were therefore further adjusted for in the multivariable Cox models. GERD indicates gastroesophageal reflux disease; GI, gastrointestinal; IBD, inflammatory bowel disease; MASLD, metabolic dysfunction–associated steatotic liver disease; UC, ulcerative colitis

We identified 16 387 patients with IBD (mean [SD] age, 74.73 [6.79] years; 9861 [60.18%] female) who were continuously enrolled in Medicare and initiated treatment with either immunomodulators, biologics, or 5-ASA (eFigure 1 in Supplement 1). Following PS matching, 4384 patients were included in the immunomodulators vs 5-ASA cohort and 3064 patients in the biologics vs 5-ASA cohort. Prior to matching, both immunomodulators vs 5-ASA and biologics vs 5-ASA cohorts predominantly included female participants (9171 [60.30%] female and 8919 [59.91%] female) with mean (SD) ages of 74.86 (6.86) years and 74.80 (6.83) years, respectively. There were 503 Black patients (3.31%), 14 023 White patients (92.20%), and 683 patients (4.49%) with other race and ethnicity in the immunomodulators vs 5-ASA cohort and 480 Black patients (3.22%), 13 733 White patients (92.24%), and 675 patient (4.53%) with other race and ethnicity in the biologics vs 5-ASA cohort. Treatment groups differed notably in cardiovascular comorbidities, frailty, and IBD-related characteristics (Figure 1). After PS matching, these imbalances were largely eliminated, and most covariates achieved SMDs less than 0.1 (eTable 1 and eTable 2 in Supplement 1). In the biologics vs 5-ASA comparison, rheumatoid arthritis and bowel-related complications remained slightly imbalanced and were therefore further adjusted for in the multivariable Cox models.

In the unmatched cohort, 70 patients in the immunomodulator group (4.67%) and 637 patients in the 5-ASA group (4.65%) experienced MACE events, corresponding to crude incidence rates of 4.58 and 5.02 events per 100 person-years, respectively (IRR, 0.91 [95% CI, 0.71-1.17]). Rates of myocardial infarction, stroke, and all-cause mortality were similar between groups (Table 1). After PS matching, patients in the immunomodulator group had a MACE incidence rate of 4.08 events per 100 person-years compared with 4.93 events per 100 person-years among patients in the 5-ASA group (IRR, 0.83 [95% CI, 0.60-1.15]). Similarly, in the unmatched biologics vs 5-ASA cohort, 65 patients (5.52%) vs 637 patients (4.65%) experienced MACE events (4.66 vs 5.02 events per 100 person-years; IRR, 0.93 [95% CI, 0.72-1.20]), and 42 patients (5.48%) vs 97 patients (4.22%) experienced MACE events in the matched cohort (4.43 vs 5.09 events per 100 person-years; IRR, 0.87 [95% CI, 0.61-1.25]). For both immunomodulators and biologics, point estimates for myocardial infarction, stroke, and all-cause mortality were below 1 but not statistically significant.

**Table 1.** Comparison of IRs for MACE Outcomes Between Immunomodulators and 5-ASA Groups Before and After Propensity Score Matching

Outcome	Immunomodulators group					5-ASA group					
	Patients, No. (%)	IR per 100 PY	Exposure time, y	Follow-up, mean (SD), d		Patients, No. (%)	IR per 100 PY	Exposure time, y	Follow-up, mean (SD), d		IRR (95% CI)
				With event	Without event				With event	Without event	
**Before matching** ^a^											
MACE	70 (4.67)	4.58	1529.86	357.56 (287.26)	373.51 (358.74)	637 (4.65)	5.02	12 688.81	323.72 (291.80)	338.74 (350.02)	0.91 (0.71-1.17)
Myocardial infarction	<11^b^	0.65	NA^b^	445.10 (295.69)	373.06 (356.38)	86 (0.63)	0.67	12 758.17	311.86 (300.82)	340.07 (348.88)	0.97 (0.50-1.86)
Stroke	<11^b^	0.58	NA^b^	318.33 (234.37)	375.28 (357.80)	103 (0.75)	0.81	12 743.43	304.39 (255.35)	339.77 (348.93)	0.72 (0.37-1.43)
All-cause mortality	57 (3.80)	3.70	1541.92	357.32 (291.22)	376.44 (359.90)	479 (3.49)	3.74	12 812.79	334.81 (299.96)	341.58 (351.05)	0.99 (0.75-1.30)
**After matching** ^c^											
MACE	48 (4.38)	4.08	1175.62	352.00 (285.74)	393.61 (369.31)	146 (4.44)	4.93	2963.49	310.50 (288.36)	330.07 (344.85)	0.83 (0.60-1.15)
Myocardial infarction	<11^b^	0.68	NA^b^	501.50 (302.61)	391.85 (366.79)	24 (0.73)	0.80	2985.2	226.13 (214.23)	332.39 (344.88)	0.84 (0.38-1.88)
Stroke	<11^b^	0.68	NA^b^	313.75 (250.12)	394.62 (368.42)	21 (0.64)	0.70	2982.35	272.76 (258.79)	331.67 (343.81)	0.96 (0.43-2.17)
All-cause mortality	38 (3.47)	3.21	1184.94	342.84 (282.04)	396.76 (370.68)	105 (3.19)	3.50	3004.05	347.15 (314.43)	333.26 (345.99)	0.92 (0.63-1.33)

Abbreviations: 5-ASA indicates 5-aminosalicylic acid; IR, incidence rate; IRR, incidence rate ratio; MACE, major adverse cardiovascular event; PY, person-years.

^a^ Including 1499 patients in the immunomodulators group and 13 710 patients in the 5-ASA group.

^b^ Cell counts reported as <11 reflect small numbers suppressed in accordance with data privacy and confidentiality policies.

^c^ Including 1096 patients in the immunomodulators group and 3288 patients in the 5-ASA group.

**Figure 2.**
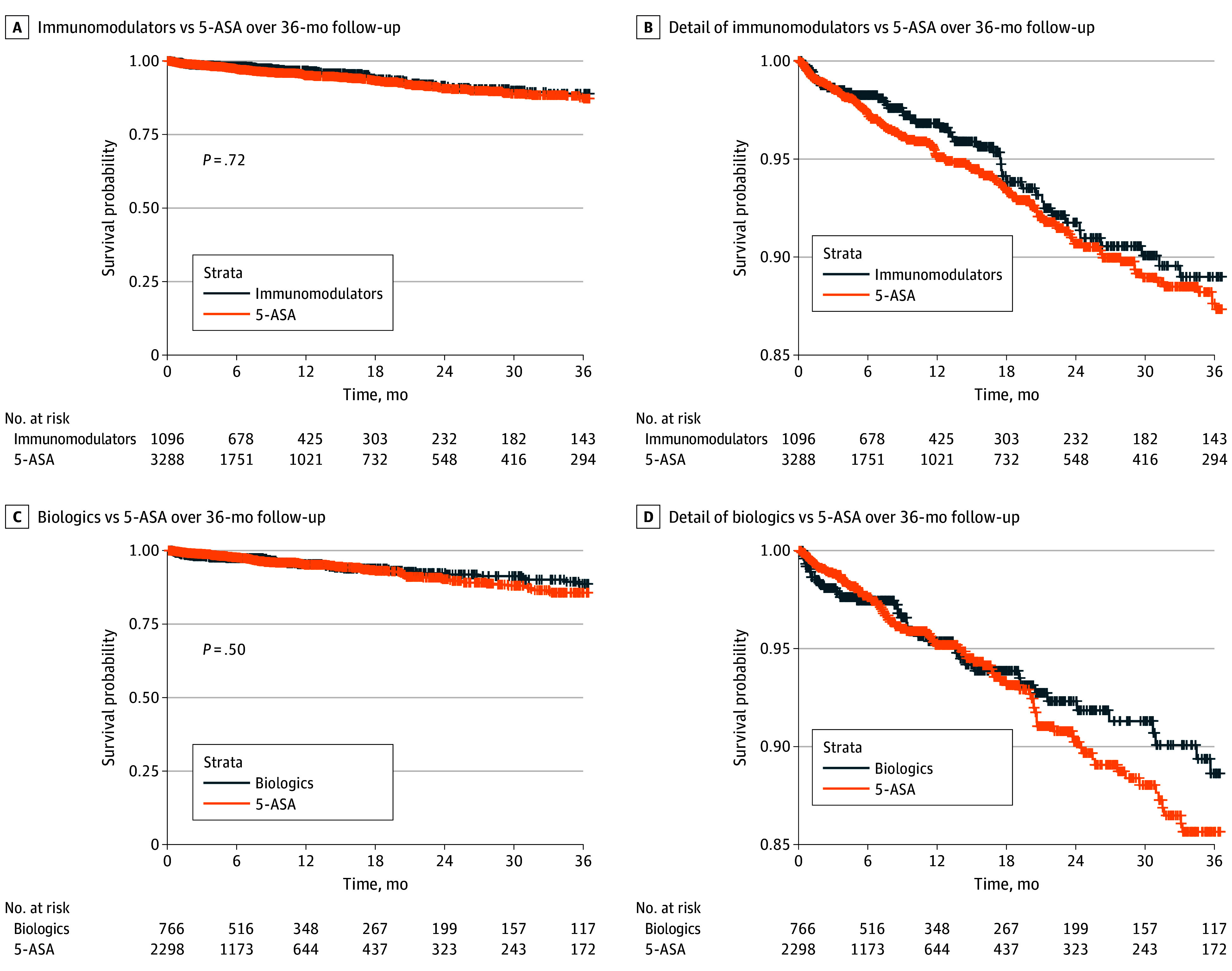
Kaplan-Meier Curves for Time to Major Adverse Cardiovascular Events Between Inflammatory Bowel Disease Therapies in the Primary Analysis 5-ASA indicates 5-aminosalicylic acid.

The median (IQR) follow-up times were 250.50 (86.00-608.00) days vs 180.00 (60.00-477.00) days in the immunomodulators vs 5-ASA cohort, and 316.50 (135.00-730.00) days vs 180.00 (60.00-414.00) days in the biologics vs 5-ASA cohort. Patients receiving immunomodulators or biologics did not have statistically significant differences in cardiovascular outcomes compared with patients using 5-ASA (Figure 2, Table 1, and Table 2). Schoenfeld residual-based tests did not indicate any significant violations of the proportional hazards assumption. There were no significantly significant differences in risk of MACE in the immunomodulators vs 5-ASA cohort (HR, 0.84 [95% CI, 0.61-1.17]) or biologics vs 5-ASA (HR, 0.86 [95% CI, 0.59-1.24]). Kaplan-Meier curves did not differ between groups based on stratified log-rank tests (immunomodulators vs 5-ASA: *P* = .72; biologics vs 5-ASA: *P* = .50). After 3 years of follow-up, the standardized cumulative incidence of MACE was 10.93% and 12.84% in the immunomodulators and 5-ASA groups, respectively, and 12.27% and 13.72% in the biologics and 5-ASA groups, respectively (eFigure 2 in Supplement 1). Similar trends were observed for individual components of MACE, with no statistically significant associations for myocardial infarction, stroke, or all-cause mortality.

**Table 2.** Comparison of IRs for MACE Outcomes Between Biologics and 5-ASA Groups Before and After Propensity Score Matching

Outcome	Biologics group					5-ASA group					
	Patients, No. (%)	IR per 100 PY	Exposure time, y	Follow-up, mean (SD), d		Patients, No. (%)	IR per 100 PY	Exposure, y	Follow-up, mean (SD), d		IRR (95% CI)
				With event	Without event				With event	Without event	
**Before matching** ^a^											
MACE	65 (5.52)	4.66	1393.49	282.34 (303.09)	440.81 (361.85)	637 (4.65)	5.02	12 688.81	323.72 (291.80)	338.74 (350.02)	0.93 (0.72-1.20)
Myocardial infarction	<11^b^	0.50	NA^b^	250.00 (274.17)	434.56 (362.01)	86 (0.63)	0.67	12 758.17	311.86 (300.82)	340.07 (348.88)	0.74 (0.34-1.60)
Stroke	<11^b^	0.43	NA^b^	627.00 (299.45)	432.69 (360.77)	103 (0.75)	0.81	12 743.43	304.39 (255.35)	339.77 (348.93)	0.53 (0.23-1.21)
All-cause mortality	55 (4.67)	3.92	1403.23	267.58 (308.66)	443.29 (362.35)	479 (3.49)	3.74	12 812.79	334.81 (299.96)	341.58 (351.05)	1.05 (0.79-1.39)
**After matching** ^c^											
MACE	42 (5.48)	4.43	947.74	319.21 (311.03)	459.61 (374.55)	97 (4.22)	5.09	1905.69	334.75 (291.37)	301.49 (327.10)	0.87 (0.61-1.25)
Myocardial infarction	<11^b^	0.63	NA^b^	283.00 (284.70)	455.20 (374.41)	15 (0.65)	0.78	1917.15	283.47 (215.43)	304.86 (327.26)	0.81 (0.31-2.08)
Stroke	<11^b^	0.42	NA^b^	515.50 (304.39)	453.35 (373.24)	14 (0.61)	0.73	1914.56	315.43 (280.32)	304.24 (326.86)	0.57 (0.19-1.75)
All-cause mortality	34 (4.44)	3.56	955.52	312.97 (331.51)	462.24 (374.92)	73 (3.18)	3.79	1926.02	357.78 (309.62)	304.43 (327.97)	0.94 (0.62-1.41)

Abbreviations: 5-ASA indicates 5-aminosalicylic acid; IR, incidence rate; IRR, incidence rate ratio; MACE, major adverse cardiovascular event; PY, person-years.

^a^ Including 1178 patients in the immunomodulators group and 13 710 patients in the 5-ASA group.

^b^ Cell counts reported as <11 reflect small numbers suppressed in accordance with data privacy and confidentiality policies.

^c^ Including 766 patients in the immunomodulators group and 2298 patients in the 5-ASA group.

**Figure 3.**
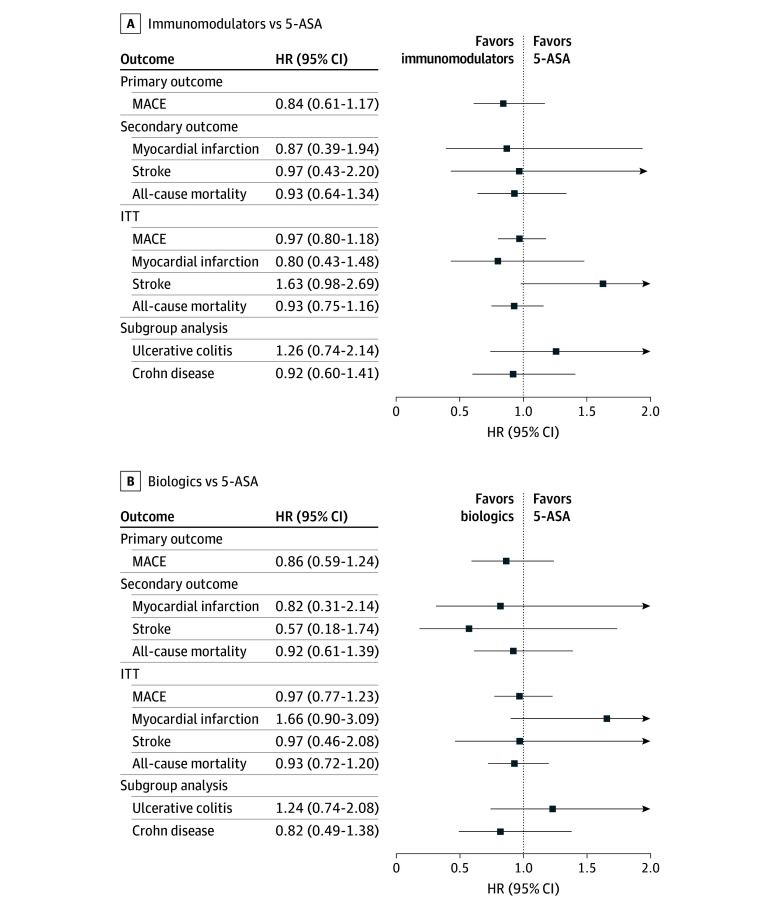
Dot Plots of Primary, Secondary, and Subgroup Analyses for the Association of Inflammatory Bowel Disease Treatments With Risk of Major Adverse Cardiovascular Events (MACE) 5-ASA indicates 5-aminosalicylic acid; HR, hazard ratio; ITT, intention to treat.

In subgroup analyses stratified by IBD subtype, subgroup-specific HRs for MACE were estimated for UC and CD (Figure 3). After rematching, among patients with UC, immunomodulator or biologic use showed no association with MACE compared with 5-ASA (immunomodulators vs 5-ASA: HR, 1.26 [95% CI, 0.74-2.14]; biologics vs 5-ASA: HR, 1.24 [95% CI, 0.74-2.08]). Among patients with CD, neither immunomodulators nor biologics therapy demonstrated significant associations with MACE (immunomodulators vs 5-ASA: HR, 0.92 [95% CI, 0.60-1.40]; biologics vs 5-ASA: HR, 0.82 [95% CI, 0.49-1.38]). In the primary PS-matched cohort, we additionally tested for interaction by including a cross-product term between treatment group and IBD subtype in the Cox model. The interaction term was not statistically significant for immunomodulators vs 5-ASA (*P* for interaction = .62) or for biologics vs 5-ASA (*P* for interaction = .13), indicating no evidence of association modification by IBD subtype.

Findings were broadly consistent across sensitivity analyses (Figure 3; eFigure 3 in Supplement 1). For the intention-to-treat approach, the were no significant differences in risk of MACE (immunomodulators vs 5-ASA: HR, 0.97 [95% CI, 0.80-1.18]; biologics vs 5-ASA: HR, 0.97 [95% CI, 0.77-1.23]). When all-cause mortality was restricted to cardiovascular-related death, the point estimates remained directionally similar (immunomodulators vs 5-ASA: HR, 0.82 [95% CI, 0.57-1.18]; biologics vs 5-ASA: HR, 0.70 [95% CI, 0.44-1.14]). Incorporating a 6-month latent period yielded attenuated estimates (immunomodulators vs 5-ASA: HR, 1.07 [95% CI, 0.81-1.41]; biologics vs 5-ASA: HR, 0.98 [95% CI, 0.70-1.36]), and analysis using a 1-month latent period also produced no significant associations (immunomodulators vs 5-ASA: HR, 0.90 [95% CI, 0.67-1.23]; biologics vs 5-ASA: HR, 0.66 [95% CI, 0.45-0.98]). With inverse probability censoring weight applied, both immunomodulators and biologics again demonstrated no associations with MACE (immunomodulators vs 5-ASA: HR, 0.55 [95% CI, 0.43-0.71]; biologics vs 5-ASA: HR, 0.62 [95% CI, 0.44-0.88]). In the negative control analysis using injury-related emergency department visits, no significant associations were observed (immunomodulators vs 5-ASA: HR, 0.98 [95% CI, 0.74-1.31]; biologics vs 5-ASA: HR, 0.91 [95% CI, 0.62-1.34]).

## Discussion

In this comparative effectiveness analysis of patients with IBD aged older than 65 years, we investigated the association of the use of immunomodulators or biologics with the risk of MACE compared with 5-ASA. Across all comparisons, point estimates for MACE comparing immunomodulators or biologics with 5-ASA were generally less than 1, although they were not statistically significant. Similar patterns were observed across multiple sensitivity analyses. Notably, applying a 6-month latent period attenuated the estimates, likely because this lag window was disproportionately long relative to the short systemic activity of 5-ASA.^31^ In subgroup analyses stratified by IBD subtype, estimates differed in direction between UC and CD; however, no statistically significant differences were observed by IBD subtypes. These exploratory findings may be considered in the context of prior literature suggesting differences in cardiovascular risk profiles between UC and CD related to systemic inflammation, endothelial dysfunction, and prothrombotic tendencies.^11,33^

The patterns we observed in this study are consistent with the pharmacologic and pathological mechanisms demonstrated in prior literature. IBD is characterized by persistent cytokine activation that promotes endothelial dysfunction, arterial stiffness, and a prothrombotic milieu,^34,35^ a central mechanism in CVD. Therefore, therapies that effectively suppress systemic inflammation may mitigate downstream proatherogenic effects. Immunomodulators, such as methotrexate, have been shown to suppress T-cell proliferation and inflammatory cytokines and to exert vascular benefits through adenosine-mediated anti-inflammatory pathways, including improved endothelial function, slowed atherosclerosis, and enhanced cholesterol transport.^36,37^ Similarly, some biologic therapies, particularly anti–TNF-α agents, directly neutralize key cytokines.^38,39,40^ Anti–IL-12 and -23 and anti-integrin agents may modulate upstream immune signaling and leukocyte trafficking, attenuating systemic immune activation and cardiovascular complications.^41,42^ In contrast, certain therapies may deteriorate cardiovascular functions. Some immunomodulators (eg, cyclosporine, tacrolimus) can induce hypertension through renal and vascular vasoconstriction and sodium retention^43^; worsen dyslipidemia by elevating low-density lipoprotein, very low–density lipoprotein, and triglycerides^44^; and contribute to glucose dysregulation.^45^ These hemodynamic and metabolic perturbations, along with endothelial dysfunction and vascular remodeling, represent mechanistic explanations for the increased incidence of MACE end points in individuals with IBD.^46^ Experimental evidence from in vitro vascular smooth muscle cell studies and ex vivo rat aortic ring models also suggests that thiopurines promote vascular smooth muscle cell osteogenic transdifferentiation, oxidative stress, and vascular calcification related to arterial stiffness and adverse cardiovascular outcomes.^47^ Collectively, these mechanisms provide a biological rationale for why therapies could plausibly influence cardiovascular outcomes, whereas 5-ASA exerts mainly local intestinal effects with limited systemic anti-inflammation effects.^5^

Prior studies in other inflammatory disease populations have reported associations between inflammation-modifying therapies and cardiovascular outcomes. In a meta-analysis of patients with rheumatoid arthritis, methotrexate use was associated with a 28% reduced risk of all cardiovascular events,^37^ and another study reported a 70% reduction in cardiovascular mortality among individuals using methotrexate.^48^ Similarly, a multicenter study using the TriNetX database in IBD found advanced biologic therapies (ie, anti-TNFs, vedolizumab, and ustekinumab) were associated with significantly reduced composite atherosclerotic CVD events compared with conventional therapy, with probabilities of 13.0% vs 17.9% by 3 years and 15.0% vs 21.2% by 5 years, along with 28% and 29% lower risks of myocardial infarction and stroke, respectively.^49^

However, not all studies have demonstrated favorable associations. A network meta-analysis of 36 RCTs (including 126 961 patients) across immune-mediated inflammatory diseases found anti-TNF and anti–IL-12 and -23 to be associated with higher odds of MACE vs placebo.^24^ This discrepancy may reflect the limited size and composition of trial populations, as IBD studies often enroll younger patients with low risk and have short follow-up, limiting the ability to detect cardiovascular events. A 2022 study^50^ reported higher venous thromboembolism risk associated with immunomodulator use, likely due to confounding by indication and population differences, whereas our Medicare cohort with higher baseline cardiovascular risk better captured long-term cardiovascular outcomes. The discrepancy may also stem from methodological and outcome differences. The nested case-control analysis by Fujiya et al^50^ compared individuals using immunomodulators with nonusers and assessed acute, hospitalization-related venous thromboembolism events, whereas our PS-matched active-comparator study evaluated long-term cardiovascular outcomes associated with by chronic systemic inflammation.

### Limitations

Our study has several limitations. First, the Medicare claims data lack biomarkers. Information such as IBD activity indices, inflammatory markers, and body mass index are not captured in claims. It is possible that patients using immunomodulators or biologics had lower disease activity (by indication of achieving remission) or were treated by subspecialists (ie, gastroenterologists) who also more aggressively managed cardiovascular risk factors. However, we attempted to proxy some of this by adjusting for steroid use, health care utilization, and time from diagnosis of IBD to drug initiation. Second, our primary outcome, MACE, is a surrogate composite end point that may mask heterogeneity in the risk of individual components. Nevertheless, MACE remains a well-validated and widely used end point across clinical trials and observational studies.^28^ Additionally, the median follow-up time was relatively short (approximately 6-10 months). Given that many cardiovascular outcomes develop over longer periods, the limited duration of follow-up may have reduced our ability to precisely detect long-term effects of treatment. Furthermore, although we adjusted for extensive clinical covariates, unmeasured or misclassified confounders (eg, over-the-counter drug use, socioeconomic status) may still introduce bias. Nonetheless, our negative control analyses did not demonstrate spurious associations, indicating that any residual confounding is unlikely to substantially alter the observed results.

## Conclusions

In this comparative effectiveness study of older adults with IBD, the use of immunomodulators or biologics was not associated with an increased risk of MACE compared with use of 5-ASA. Although no statistically significant differences in cardiovascular risk were observed, these findings provide reassurance regarding the cardiovascular safety of therapies targeting systemic inflammation in this high-risk population. Future studies integrating clinical and biomarker data are needed to elucidate the mechanisms linking anti-inflammatory treatment with cardiovascular health.

## Notes

Supplement 1. eTable 1. Comparison of baseline characteristics in Immunomodulators vs 5-ASA cohort before and after PSM eTable 2. Comparison of baseline characteristics in Biologics vs 5-ASA cohort before and after PSM eFigure 1. Study Enrollment Flowchart eFigure 2. Cumulative incidence of MACE between IBD treatments in the primary analysis eFigure 3. Sensitivity analyses for the association between IBD treatments and the risk of MACE among IBD patients

Supplement 2. Data Sharing Statement
