# Supplementary material for: Immunomodulators, Biologics, and 5-ASA for Inflammatory Bowel Disease and Major Adverse Cardiovascular Events in Older Adults
Source: JAMA Netw Open. 2026 Apr 29;9(4):e269091. doi: 10.1001/jamanetworkopen.2026.9091 (PMC13129880; doi:10.1001/jamanetworkopen.2026.9091)
Supplement: Supplement 1. — eTable 1. Comparison of baseline characteristics in Immunomodulators vs 5-ASA cohort before and after PSM eTable 2. Comparison of baseline characteristics in Biologics vs 5-ASA cohort before and after PSM eFigure 1. Study Enrollment Flowchart eFigure 2. Cumulative incidence of MACE between IBD treatments in the primary analysis eFigure 3. Sensitivity analyses for the association between IBD treatments and the risk of MACE among IBD patients [file jamanetwopen-e269091-s001.pdf]

Supplemental Online Content

Jian Q, Chaudhry NA, Du F, Zimmermann E, Jiao T. Immunomodulators, biologics, and 5-ASA inflammatory bowel disease and major adverse cardiovascular events in older adults. *JAMA Netw Open*. 2026;9(4):e269091.
doi:10.1001/jamanetworkopen.2026.9091

eTable 1. Comparison of baseline characteristics in Immunomodulators vs 5-ASA cohort before and after PSM

eTable 2. Comparison of baseline characteristics in Biologics vs 5-ASA cohort before and after PSM

eFigure 1. Study Enrollment Flowchart

eFigure 2. Cumulative incidence of MACE between IBD treatments in the primary analysis

eFigure 3. Sensitivity analyses for the association between IBD treatments and the risk of MACE among IBD patients

This supplemental material has been provided by the authors to give readers additional information about their work.

© 2026 Jian Q et al. *JAMA Network Open*.

eTable 1. Comparison of baseline characteristics in Immunomodulators vs. 5-ASA cohort before and after PSM.

	Before match			After match		
	All (n=15,209)	IM (n=1,499)	5-ASA (n=13,710)	ALL (n=4,384)	IM (n=1,096)	5-ASA (n=3,288)
**Demographics**						
**Age, years**						
Mean (SD)	74.86 (6.86)	73.97 (6.27)	74.96 (6.91)	74.05 (6.43)	73.96 (6.37)	74.08 (6.46)
[65,70)	4,539 (29.84%)	498 (33.22%)	4,041 (29.47%)	1,482 (33.80%)	372 (33.94%)	1,110 (33.76%)
[70,75)	4,252 (27.96%)	436 (29.09%)	3,816 (27.83%)	1,256 (28.65%)	309 (28.19%)	947 (28.80%)
[75,80)	3,009 (19.78%)	296 (19.75%)	2,713 (19.79%)	842 (19.21%)	213 (19.43%)	629 (19.13%)
≥80	3,409 (22.41%)	269 (17.95%)	3,140 (22.90%)	804 (18.34%)	202 (18.43%)	602 (18.31%)
**Female gender**	9,171 (60.30%)	492 (62.84%)	8,229 (60.02%)	2,580 (58.85%)	651 (59.40%)	1,929 (58.67%)
**Race/ethnicity**						
White	14,023 (92.20%)	1,384 (92.33%)	12,639 (92.19%)	4,082 (93.11%)	1,020 (93.07%)	3,062 (93.13%)
Black	503 (3.31%)	48 (3.30%)	455 (3.32%)	142 (3.24%)	33 (3.01%)	109 (3.32%)
Other	683 (4.49%)	67 (4.47%)	616 (4.49%)	160 (3.65%)	43 (3.92%)	117 (3.56%)
**Life style factor**						
**Tobacco use**						
Current	777 (5.11%)	105 (7.00%)	642 (4.90%)	292 (6.66%)	75 (6.84%)	217 (6.60%)
Former	2,003 (13.17%	215 (14.34%)	1,788 (13.04%)	557 (12.71%)	138 (12.59%)	419 (12.74%)
Never	12,429 (81.72%)	1,179 (78.65%)	11,250 (82.06%)	3,535 (80.63%)	883 (80.57%)	2,652 (80.66%)
Alcohol disorder	229 (1.51%)	32 (2.13%)	197 (1.44%)	65 (1.48%)	19 (1.73%)	46 (1.40%)
**Medical history**						
Hypertension	10,651 (70.03%)	1,058 (70.58%)	9,593 (69.97%)	2,951 (67.31%)	750 (68.43%)	2,201 (66.94%)
Type II diabetes	3,756 (24.70%)	409 (27.28%)	3,347 (24.41%)	1,087 (24.79%)	275 (25.09%)	812 (24.70%)
CKD	1,973 (12.97%)	264 (17.61%)	1,709 (12.47%)	625 (14.26%)	157 (14.32%)	468 (14.23%)
Hyperlipidemia	10,065 (66.18%)	936 (62.44%)	9,129 (66.59%)	2,706 (61.72%)	680 (62.04%)	2,026 (61.62%)
Coronary Artery Disease (CAD)	3,308 (21.75%)	336 (22.41%)	2,972 (21.68%)	910 (20.76%)	237 (21.62%)	673 (20.47%)
Metabolic Dysfunction-associated Steatotic Liver Disease (MASLD)	351 (2.31%)	45 (3.00%)	306 (2.23%)	110 (2.51%)	28 (2.55%)	82 (2.49%)
Rheumatoid Arthritis (RA)	692 (4.55%)	313 (20.88%)	379 (2.76%)	79 (1.80%)	21 (1.92%)	58 (1.76%)
Asthma	1,239 (8.15%)	139 (9.27%)	1,100 (8.02%)	352 (8.03%)	92 (8.39%)	260 (7.91%)
Chronic Obstructive Pulmonary Disease (COPD)	1,906 (12.53%)	205 (13.68%)	1,701 (12.41%)	557 (12.71%)	134 (12.23%)	423 (12.86%)
Organ transplant	357 (2.35%)	99 (6.60%)	258 (1.88%)	122 (2.78%)	30 (2.74%)	92 (2.80%)

© 2026 Jian Q et al. *JAMA Network Open*.

Obesity	1,594 (10.48%)	174 (11.61%)	1,420 (10.36%)	482 (10.99%)	118 (10.77%)	364 (11.07%)
Gastroesophageal Reflux Disease (GERD)	4,503 (29.61%)	511 (34.09%)	3,992 (29.12%)	1,357 (30.95%)	343 (31.30%)	1,014 (30.84%)
Osteoarthritis	2,757 (18.13%)	346 (23.08%)	2,411 (17.59%)	723 (16.49%)	218 (19.89%)	505 (15.36%)
Anemia	4,242 (27.89%)	519 (34.62%)	3,723 (27.16%)	1,270 (28,97%)	335 (30.57%)	935 (28.44%)
Thyroid disorder	3,836 (25.22%)	406 (27.08%)	3,430 (25.02%)	1,088 (24.82%)	274 (25.00%)	814 (24.76%)
Depression	1,624 (10.68%)	201 (13.41%)	1,423 (10.38%)	472 (10.77%)	120 (10.95%)	352 (10.71%)
Anxiety	2,125 (13.97%)	250 (16.68%)	1,875 (13.68%)	631 (14.39%)	156 (14.23%)	475 (14.45%)
Bipolar Disorder	364 (2.40%)	34 (2.27%)	331 (2.41%)	99 (2.26%)	25 (2.28%)	74 (2.25%)
Malnutrition	1,589 (10.45%)	215 (14.34%)	1,374 (10.02%)	521 (11.88%)	135 (12.32%)	386 (11.74%)
IBD-related surgery	181 (1.19%)	38 (2.54%)	143 (1.04%)	71 (1.62%)	15 (1.37%)	56 (1.70%)
Non-malignant skin cancer	1,408 (9.26%)	203 (13.54%)	1,205 (8.79%)	545 (12.43%)	126 (11.50%)	419 (12.74%)
**IBD complications**						
Bowel related complications	2,156 (14.18%)	163 (10.87%)	1,993 (14.54%)	459 (10.47%)	120 (10.95%)	339 (10.31%)
Liver and hepatobiliary complications	559 (3.68%)	84 (5.60%)	475 (3.46%)	194 (4.43%)	51 (4.65%)	143 (4.35%)
Hematological complications	1,096 (7.21%)	154 (10.27%)	942 (6.87%)	379 (8.65%)	102 (9.31%)	277 (8.42%)
Dermatological & musculoskeletal complications	2,002 (13.16%)	355 (23.68%)	1,647 (12.01%)	644 (14.69%)	217 (19.80%)	427 (12.99%)
Renal and urinary tract complications	535 (3.52%)	68 (4.54%)	467 (3.41%)	173 (3.95%)	42 (3.83%)	131 (3.98%)
Infections complications	1,397 (9.19%)	206 (13.74%)	1,191 (8.69%)	438 (9.99%)	109 (9.95%)	329 (10.01%)
Complications of autoimmune pancreatitis	197 (1.30%)	25 (1.67%)	172 (1.25%)	70 (1.60%)	13 (1.19%)	57 (1.73%)
GI bleeding	2,323 (15.27%)	135 (9.01%)	2,188 (15.96%)	396 (9.03%)	102 (9.31%)	294 (8.94%)
**Medications**						
Steroid use	4,719 (31.03%)	491 (32.76%)	4,228 (30.84%)	1,331 (30.36%)	341 (31.11%)	990 (30.11%)
Statin use	7,723 (50.78%)	679 (45.30%)	7,044 (51.38%)	2,019 (46.05%)	498 (45.44%)	1,521 (46.26%)
**Others**						
**QRISK score**						
<10%	651 (4.28%)	52 (3.47%)	599 (4.37%)	187 (4.27%)	48 (4.38%)	139 (4.23%)
10%-20%	4,138 (27.21%)	399 (26.62%)	3,739 (27.27%)	1,271 (28.99%)	308 (28.10%)	963 (29.29%)
20%-30%	3,789 (24.91%)	380 (25.35%)	3,409 (24.87%)	1,168 (26.64%)	284 (25.91%)	884 (26.89%)
30%+	6,631 (43.60%)	668 (44.56%)	5,963 (43.49%)	1,758 (40.10%)	456 (41.61%)	1,302 (39.60%)
**Frailty score**						
<0.2	12,586 (82.75%)	1,222 (81.52%)	11,364 (82.89%)	3,731 (85.10%)	933 (85.13%)	2,798 (85.10%)
0.2–0.35	1,721 (11.32%)	194 (12.94%)	1,527 (11.14%)	461 (10.52%)	116 (10.58%)	345 (10.49%)

© 2026 Jian Q et al. *JAMA Network Open*.

≥0.35	902 (5.93%)	83 (5.54%)	819 (5.97%)	192 (4.38%)	47 (4.29%)	145 (4.41%)
**Charlson Comorbidity Index (CCI)**						
0-1	8,892 (58.47%)	752 (50.17%)	8,140 (59.37%)	2,539 (57.92%)	634 (57.85%)	1,905 (57.94%)
1+	6,317 (41.53%)	747 (49.83%)	5,570 (40.63%)	1,845 (42.08%)	462 (42.15%)	1,383 (42.06%)
**IBD duration (month)**						
<1	4,945 (32.51%)	255 (17.01%)	4,690 (34.21%)	866 (19.75%)	220 (20.07%)	646 (19.65%)
1-3	5,319 (34.97%)	517 (34.39%)	4,802 (35.03%)	1,557 (35.52%)	378 (34.49%)	1,179 (35.86%)
3-6	1,754 (11.53%)	196 (13.08%)	1,558 (11.36%)	562 (12.82%)	140 (12.77%)	422 (12.83%)
6+	3,191 (20.98%)	531 (35.42%)	2,660 (19.40%)	1,399 (31.91%)	358 (32.66%)	1,041 (31.66%)
**IBD category**						
UC	10,461 (68.78%)	646 (43.10%)	9,815 (71.59%)	1,868 (42.61%)	467 (42.61%)	1,401 (42.61%)
CD	4,748 (31.22%)	853 (56.90%)	3,895 (28.41%)	2,516 (57.39%)	629 (57.39%)	1,887 (57.39%)
**Index year**						
2012	1,954 (12.85%)	220 (14.68%)	1,734 (12.65%)	684 (15.60%)	185 (16.88%)	499 (15.18%)
2013	1,368 (8.99%)	114 (7.61%)	1,254 (9.15%)	353 (8.05%)	93 (8.49%)	260 (7.91%)
2014	1,117 (7.34%)	78 (5.20%)	1,039 (7.58%)	237 (5.41%)	62 (5.66%)	175 (5.32%)
2015	835 (5.49%)	68 (4.54%)	767 (5.59%)	203 (4.63%)	49 (4.47%)	154 (4.68%)
2016	773 (5.08%)	89 (5.94%)	684 (4.49%)	205 (4.68%)	46 (4.20%)	159 (4.84%)
2017	3,368 (22.14%)	298 (19.88%)	3,070 (22.39%)	967 (22.06%)	228 (20.80%)	739 (22.48%)
2018	2,329 (15.31%)	243 (16.21%)	2,086 (15.22%)	682 (15.56%)	179 (16.33%)	503 (15.30%)
2019	1,818 (11.95%)	203 (13.54%)	1,615 (11.78%)	559 (12.75%)	137 (12.50%)	422 (12.83%)
2020	1,647 (10.83%)	186 (12.41%)	1,461 (10.66%)	494 (11.27%)	117 (10.68%)	377 (11.47%)

*Continuous variables are expressed as mean ± SD. Categorical variables are expressed as number (%).

© 2026 Jian Q et al. *JAMA Network Open*.

eTable 2. Comparison of baseline characteristics in Biologics vs. 5-ASA cohort before and after PSM.

	Before match			After match		
	All (n=14,888)	Biologics (n=1,178)	5-ASA (n=13,710)	ALL (n=3,064)	Biologics (n=766)	5-ASA (n=2,298)
**Demographics**						
**Age, years**						
Mean (SD)	74.80 (6.83)	72.97 (5.52)	74.96 (6.91)	73.68 (6.01)	73.57 (5.89)	73.71 (6.05)
[65,70)	4,474 (30.05%)	433 (36.76%)	4,041 (29.47%)	1,031 (33.65%)	263 (34.33%)	768 (33.42%)
[70,75)	4,199 (28.20%)	383 (32.51%)	3,816 (27.83%)	960 (31.33%)	229 (29.90%)	731 (31.81%)
[75,80)	2,931 (19.69%)	218 (18.51%)	2,713 (19.79%)	598 (19.52%)	149 (19.45%)	449 (19.54%)
≥80	3,284 (22.06%)	144 (12.22%)	3,140 (22.90%)	475 (15.50%)	125 (16.32%)	350 (15.23%)
**Female gender**	8,919 (59.91%)	690 (58.57%)	8,229 (60.02%)	1,780 (58.09%)	444 (57.96%)	1,336 (58.14%)
**Race/ethnicity**						
White	13,733 (92.24%)	1,094 (92.87%)	12,639 (92.19%)	2,879 (93.96%)	712 (92.95%)	2,167 (94.30%)
Black	480 (3.22%)	25 (2.12%)	455 (3.32%)	79 (2.58%)	20 (2.61%)	59 (2.57%)
Other	675 (4.53%)	59 (5.01%)	616 (4.49%)	106 (3.46%)	34 (4.44%)	72 (3.13%)
**Life style factor**						
**Tobacco use**						
Current	778 (5.23%)	106 (9.00%)	672 (4.90%)	214 (6.98%)	52 (6.79%)	162 (7.05%)
Former	1,994 (13.39%)	206 (17.49%)	1,788 (13.04%)	478 (15.60%)	118 (15.40%)	360 (15.67%)
Never	12,116 (81.38%)	866 (73.51%)	11,250 (82.06%)	2,372 (77.42%)	596 (77.81%)	1,776 (77.28%)
Alcohol disorder	223 (1.50%)	26 (2.21%)	197 (1.44%)	51 (1.66%)	15 (1.96%)	36 (1.57%)
**Medical history**						
Hypertension	10,393 (69.81%)	800 (67.91%)	9,593 (69.97%)	2,091 (68.24%)	527 (68.80%)	1,564 (68.06%)
Type II diabetes	3,613 (24.27%)	266 (22.58%)	3,347 (24.41%)	678 (22.13%)	183 (23.89%)	495 (21.54%)
CKD	1,908 (12.82%)	199 (16.89%)	1,709 (12.47%)	452 (14.75%)	112 (14.62%)	340 (14.80%)
Hyperlipidemia	9,841 (66.10%)	712 (60.44%)	9,129 (66.59%)	1,933 (63.09%)	486 (63.45%)	1,447 (62.97%)
Coronary Artery Disease (CAD)	3,198 (21.48%)	226 (19.19%)	2,972 (21.68%)	626 (20.43%)	164 (21.41%)	462 (20.10%)
Metabolic Dysfunction-associated Steatotic Liver Disease (MASLD)	352 (2.36%)	46 (3.90%)	306 (2.23%)	97 (3.17%)	20 (2.61%)	77 (3.35%)
Rheumatoid Arthritis (RA)	538 (3.61%)	159 (13.50%)	379 (2.76%)	217 (7.08%)	74 (9.66%)	143 (6.22%)
Asthma	1,207 (8.11%)	107 (9.08%)	1,100 (8.02%)	281 (9.17%)	68 (8.88%)	213 (9.27%)
Chronic Obstructive Pulmonary Disease (COPD)	1,849 (12.42%)	148 (12.56%)	1,701 (12.41%)	409 (13.35%)	92 (12.01%)	317 (13.79%)
Organ transplant	302 (2.03%)	44 (3.74%)	258 (1.88%)	90 (2.94%)	21 (2.74%)	69 (3.00%)

© 2026 Jian Q et al. *JAMA Network Open*.

Obesity	1,543 (10.36%)	123 (10.44%)	1,420 (10.36%)	325 (10.61%)	85 (11.10%)	240 (10.44%)
Gastroesophageal Reflux Disease (GERD)	4,396 (29.53%)	404 (34.30%)	3,992 (29.12%)	1,006 (32.83%)	255 (33.29%)	751 (32.68%)
Osteoarthritis	2,616 (17.57%)	205 (17.40%)	2,411 (17.59%)	523 (17.07%)	141 (18.41%)	382 (16.62%)
Anemia	4,161 (27.95%)	438 (37.18%)	3,723 (27.16%)	995 (31.49%)	248 (32.38%)	717 (31.20%)
Thyroid disorder	3,703 (24.87%)	273 (23.17%)	3,430 (25.02%)	698 (22.78%)	184 (24.02%)	514 (22.37%)
Depression	1,578 (10.60%)	155 (13.16%)	1,423 (10.38%)	337 (11.00%)	85 (11.10%)	252 (10.97%)
Anxiety	2,070 (13.90%)	195 (16.55%)	1,875 (13.68%)	462 (15.08%)	114 (14.88%)	348 (15.14%)
Bipolar Disorder	352 (2.36%)	21 (1.78%)	331 (2.41%)	52 (1.70%)	16 (2.09%)	36 (1.57%)
Malnutrition	1,547 (10.39%)	173 (14.69%)	1,374 (10.02%)	403 (13.15%)	100 (13.05%)	303 (13.19%)
IBD-related surgery	207 (1.39%)	64 (5.43%)	143 (1.04%)	58 (1.89%)	15 (1.96%)	43 (1.87%)
Non-malignant skin cancer	1,331 (8.94%)	126 (10.70%)	1,205 (8.79%)	333 (10.87%)	75 (9.79%)	258 (11.23%)
**IBD complications**						
Bowel related complications	2,272 (15.26%)	279 (23.68%)	1,993 (14.54%)	525 (17.13%)	154 (20.10%)	371 (16.14%)
Liver and hepatobiliary complications	559 (3.75%)	84 (7.13%)	475 (3.46%)	134 (4.37%)	38 (4.96%)	96 (4.18%)
Hematological complications	1,134 (7.62%)	192 (16.30%)	942 (6.87%)	330 (10.77%)	75 (9.79%)	255 (11.10%)
Dermatological & musculoskeletal complications	1,904 (12.79%)	257 (21.82%)	1,647 (12.01%)	509 (16.61%)	151 (19.71%)	358 (15.58%)
Renal and urinary tract complications	561 (3.77%)	94 (7.98%)	467 (3.41%)	148 (4.83%)	37 (4.83%)	111 (4.83%)
Infections complications	1,363 (9.16%)	172 (14.60%)	1,191 (8.69%)	370 (12.08%)	92 (12.01%)	278 (12.10%)
Complications of autoimmune pancreatitis	203 (1.36%)	31 (2.63%)	172 (1.25%)	49 (1.60%)	16 (2.09%)	33 (1.44%)
GI bleeding	2,363 (15.87%)	175 (14.86%)	2,188 (15.96%)	498 (16.25%)	116 (15.14%)	382 (16.62%)
**Medications**						
Steroid use	4,613 (30.98%)	385 (32.68%)	4,228 (30.84%)	1,018 (33.22%)	246 (32.11%)	772 (33.59%)
Statin use	7,533 (50.60%)	489 (41.51%)	7,044 (51.38%)	1,397 (45.59%)	359 (46.87%)	1,038 (45.17%)
**Others**						
**QRISK score**						
<10%	648 (4.35%)	49 (4.16%)	599 (4.37%)	142 (4.63%)	37 (4.83%)	105 (4.57%)
10%-20%	4,081 (27.41%)	342 (29.03%)	3,739 (27.27%)	881 (28.75%)	211 (27.55%)	670 (29.16%)
20%-30%	3,756 (25.23%)	347 (29.46%)	3,409 (24.87%)	885 (28.88%)	220 (28.72%)	665 (28.94%)
30%+	6,403 (43.01%)	440 (37.35%)	5,963 (43.49%)	1,156 (37.73%)	298 (38.90%)	858 (37.34%)
**Frailty score**						
<0.2	12,419 (83.42%)	1,055 (89.56%)	11,364 (82.89%)	2,694 (87.92%)	667 (87.08%)	2,027 (88.21%)
0.2–0.35	1,621 (10.89%)	94 (7.98%)	1,527 (11.14%)	259 (8.45%)	80 (10.44%)	179 (7.79%)

© 2026 Jian Q et al. *JAMA Network Open*.

≥0.35	848 (5.70%)	29 (2.46%)	819 (5.97%)	111 (3.62%)	19 (2.48%)	92 (4.00%)
**Charlson Comorbidity Index (CCI)**						
0-1	8,792 (59.05%)	652 (55.35%)	8,140 (59.37%)	1,749 (57.08%)	437 (57.05%)	1,312 (57.09%)
1+	6,096 (40.95%)	526 (44.65%)	5,570 (40.63%)	1,315 (42.92%)	329 (42.95%)	986 (42.91%)
**IBD duration (month)**						
<1	4,926 (33.09%)	236 (20.03%)	4,690 (34.21%)	824 (26.89%)	203 (26.50%)	621 (27.02%)
1-3	5,076 (34.09%)	274 (23.26%)	4,802 (35.03%)	909 (29.67%)	239 (31.20%)	670 (29.16%)
3-6	1,652 (11.10%)	94 (7.98%)	1,558 (11.36%)	308 (10.05%)	77 (10.05%)	231 (10.05%)
6+	3,234 (21.72%)	574 (48.73%)	2,660 (19.40%)	1,023 (33.39%)	247 (32.25%)	776 (33.77%)
**IBD category**						
UC	10,161 (68.25%)	346 (29.37%)	9,815 (71.59%)	1,348 (43.99%)	337 (43.99%)	1,011 (43.99%)
CD	4,727 (31.75%)	832 (70.63%)	3,895 (28.41%)	1,716 (56.01%)	429 (56.01%)	1,287 (56.01%)
**Index year**						
2012	1,886 (12.67%)	152 (12.90%)	1,734 (12.65%)	479 (15.63%)	124 (16.19%)	355 (15.45%)
2013	1,268 (8.52%)	14 (1.19%)	1,254 (9.15%)	55 (1.80%)	14 (1.83%)	41 (1.78%)
2014	1,093 (7.34%)	54 (4.58%)	1,039 (7.58%)	165 (5.39%)	41 (5.35%)	124 (5.40%)
2015	801 (5.38%)	34 (2.89%)	767 (5.59%)	112 (3.66%)	32 (4.18%)	80 (3.48%)
2016	738 (4.96%)	54 (4.58%)	684 (4.99%)	154 (5.03%)	36 (4.70%)	118 (5.13%)
2017	3,285 (22.06%)	215 (18.25%)	3,070 (22.39%)	593 (19.35%)	150 (19.58%)	443 (19.28%)
2018	2,289 (15.37%)	203 (17.23%)	2,086 (15.22%)	512 (16.71%)	121 (15.80%)	391 (17.01%)
2019	1,838 (12.35%)	223 (18.93%)	1,615 (11.78%)	483 (15.76%)	121 (15.80%)	362 (15.75%)
2020	1,690 (11.35%)	229 (19.44%)	1,461 (10.66%)	511 (16.68%)	127 (16.58%)	384 (16.71%)

*Continuous variables are expressed as mean ± SD. Categorical variables are expressed as number (%).

© 2026 Jian Q et al. *JAMA Network Open*.

eFigure 1. Flowchart.

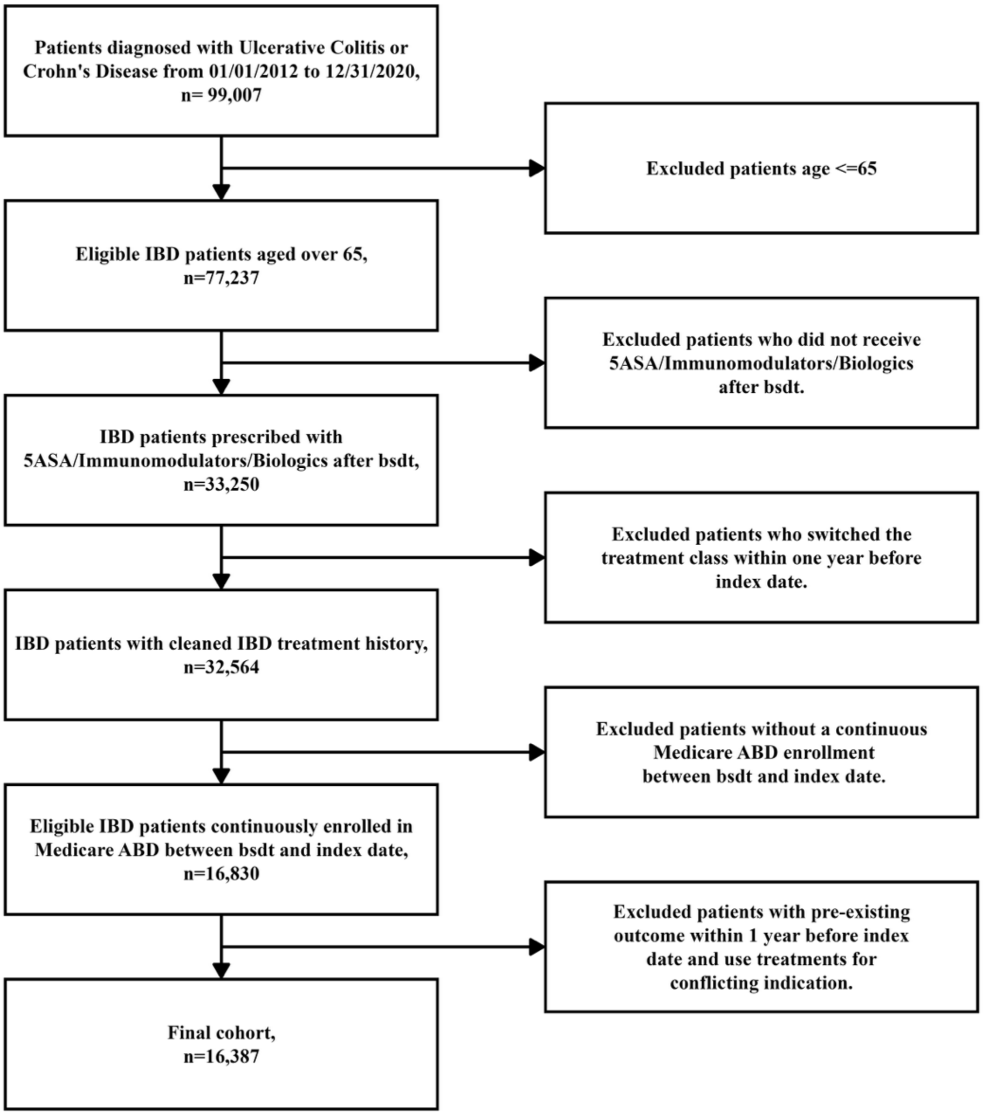

Note: bsdt denotes the base cohort entry date, defined as the date of IBD diagnosis.

© 2026 Jian Q et al. *JAMA Network Open*.

eFigure 2. Cumulative incidence of MACE between IBD treatments in the primary analysis.

a. Immunomodulators vs. 5-ASA over 36 months of follow-up.

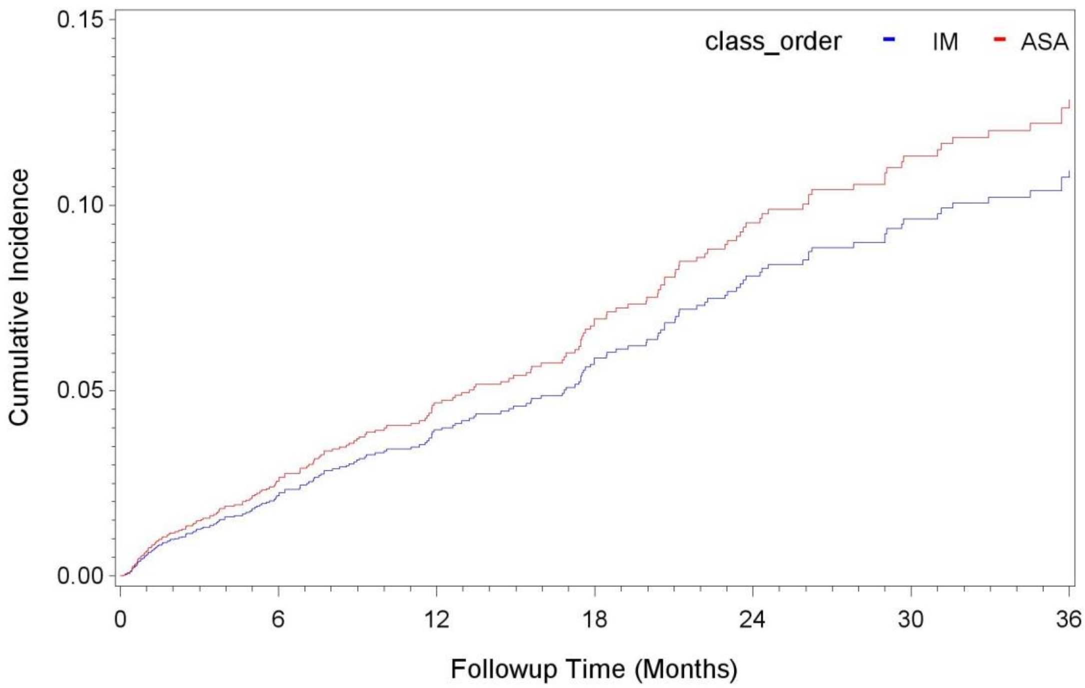

b. Biologics vs. 5-ASA over 36 months of follow-up.

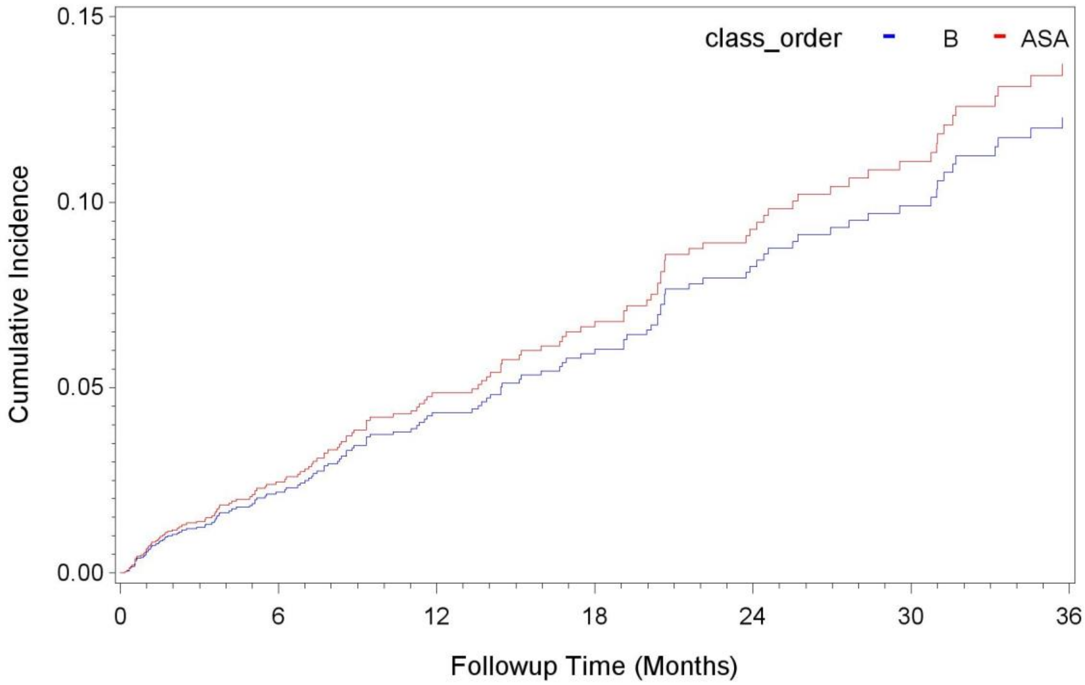

© 2026 Jian Q et al. *JAMA Network Open*.

eFigure 3. Sensitivity analyses for the association between IBD treatments and the risk of MACE among IBD patients.

a. Immunomodulators vs. 5-ASA.

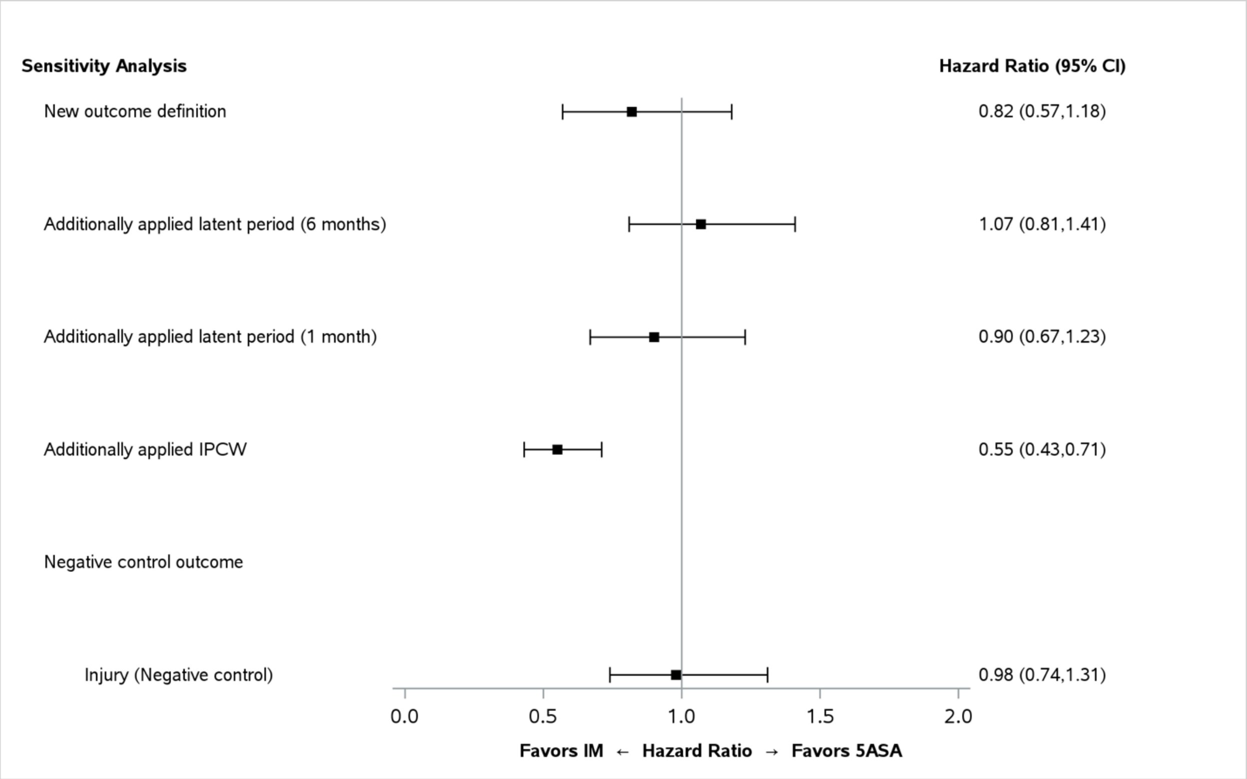

b. Biologics vs. 5-ASA.

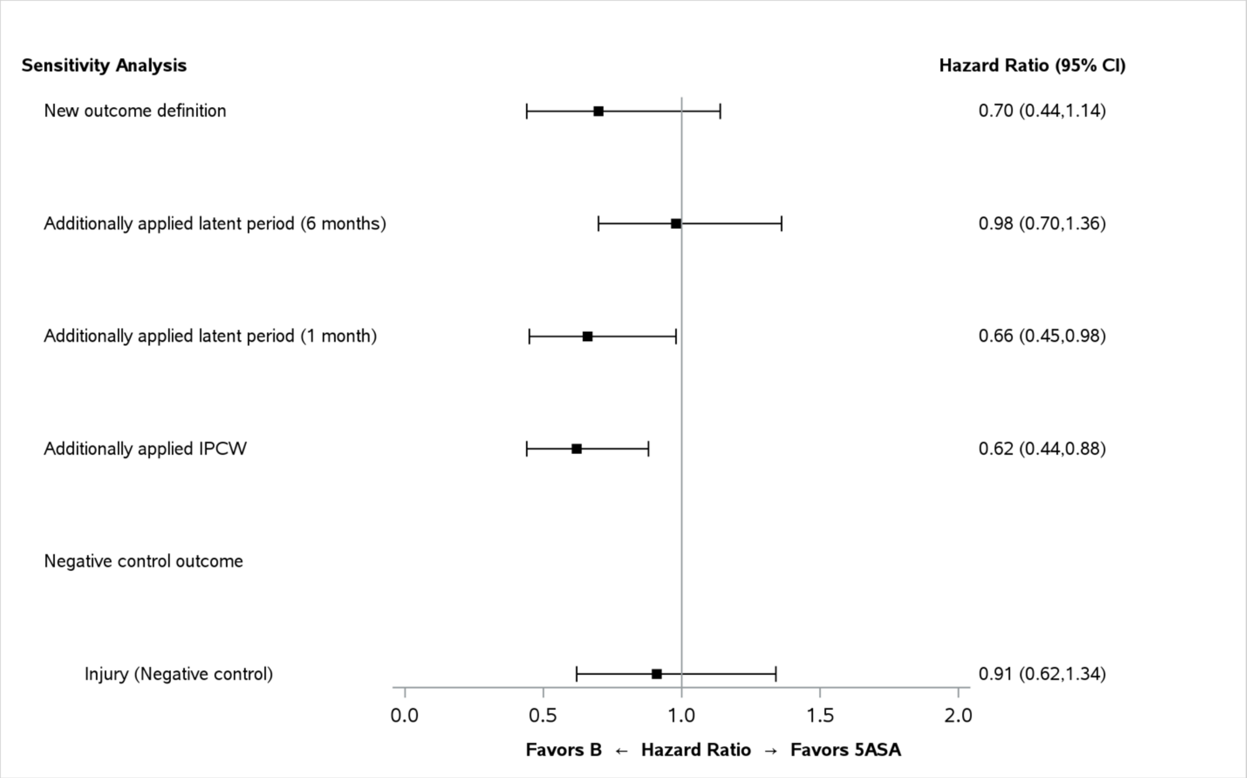

© 2026 Jian Q et al. *JAMA Network Open*.
