# Supplementary material for: Immunomodulators, Biologics, and 5-ASA for Inflammatory Bowel Disease and Major Adverse Cardiovascular Events in Older Adults
Source: JAMA Netw Open. 2026 Apr 29;9(4):e269091. doi: 10.1001/jamanetworkopen.2026.9091 (PMC13129880; doi:10.1001/jamanetworkopen.2026.9091)
Supplement: Supplement 2. — Data Sharing Statement [file jamanetwopen-e269091-s002.pdf]

Data Sharing Statement

Jian. Immunomodulators, Biologics, and 5-ASA Inflammatory Bowel Disease and Major Adverse Cardiovascular Events in Older Adults. *JAMA Netw Open*. Published April 29, 2026. doi:10.1001/jamanetworkopen.2026.9091

Data
Data available: No

Additional Information
Explanation for why data not available: The data used in this study were obtained from Medicare claims and are not publicly available. These data are subject to a data use agreement with the Centers for Medicare & Medicaid Services (CMS), which prohibits redistribution by the authors. Researchers may obtain access to the data directly from CMS upon reasonable request and approval.
